# The Effect of Age, Biological Maturation and Birth Quartile in the Kinanthropometric and Physical Fitness Differences between Male and Female Adolescent Volleyball Players

**DOI:** 10.3390/children9010058

**Published:** 2022-01-04

**Authors:** Mario Albaladejo-Saura, Raquel Vaquero-Cristóbal, Juan Alfonso García-Roca, Francisco Esparza-Ros

**Affiliations:** 1 International Kinanthropometry Chair, UCAM, Universidad Católica de Murcia, 30107 Guadalupe, Murcia, Spain; mdalbaladejosaura@ucam.edu (M.A.-S.); fesparza@ucam.edu (F.E.-R.); 2 Faculty of Sport Science, UCAM, Universidad Católica de Murcia, 30107 Guadalupe, Murcia, Spain; jagarcia@ucam.edu

**Keywords:** growth, sport performance, adolescence, anthropometry

## Abstract

Background: Differences in kinanthropometric and physical fitness performance between boys and girls usually start during adolescence, as a result of the changes in the hormonal environment that occur with the advance of age and biological maturation; Methods: A total of 96 1st Regional Division players adolescent volleyball players, 48 males, (age = 14.17 ± 1.00 years-old) and 48 females (age = 14.41 ± 1.21 years-old) underwent a kinanthropometric assessment, were asked to perform different physical fitness test and to complete a questionnaire. Chronological age, maturity offset, age at peak height velocity (APHV), and birth quartile were calculated; Results: Statistical differences were observed between male and female players in the APHV (*p* < 0.001). Male players showed higher values in the bone and muscle-related variables (*p* < 0.001–0.040), as well as in the strength and power production-related physical tests (*p* < 0.001–0.012), while the female showed higher values in the fat-related variables (*p* = 0.003–0.013), and performed better in the flexibility tests. Age, maturity offset, and birth quartile showed to have statistical influence in the differences found between sex groups; Conclusions: There is a clear influence of age and biological maturation on the differences found between sexes in adolescent volleyball players that could be taken into account regarding grouping in early stages.

## 1. Introduction

Volleyball is a highly demanding sport due to the rules of the game, characterized by constant explosive actions [1,2], which means that both physical condition and kinanthropometric variables are of great importance in performance [3]. This is why sport sciences have tried to characterize the morphological and physical requirements of elite athletes and their relationship with sporting performance [4]. In this sense, it has been observed how kinanthropometric variables such as height, arm span, and leg length could allow differentiation of high-level players [4], together with specific physical abilities such as vertical jumping and coordination in agility tests [3]. Focusing on training players, previous studies on adolescent volleyball players have found that height, arm span, and upper and lower body power are key factors for performance in both boys and girls [5,6].

However, this characterization of the requirements of athletes during puberty must be undertaken with caution due to the effect that biological maturation has been shown to have on kinanthropometric and physical fitness variables [7,8]. More specifically, there is evidence of the different rates of maturation experienced by adolescents during the process, with these differences affecting the physical manifestation of the changes inherent to adolescence [9,10]. In relation to sports performance, higher values in kinanthropometric variables and better results in the physical fitness test have been observed in those subjects who mature earlier, compared to those who mature later [8]. This could be a disadvantage for subjects who mature late, due to the traditional way of organizing sports competitions in training stages using gender and chronological age as the only criteria [11]. Not surprisingly, previous studies have pointed out that, when a player selection process takes place in collective sports, subjects whose body size is larger and whose immediate physical performance is better tend to have a higher probability of being chosen [12]. However, there are studies that show that as the maturation process continues, these characteristics could become equalized, which could mean that early recruitment is not a guarantee of future sporting success, since it is not until approximately 14 years of age that successful players show differences with respect to their peers in physical abilities [7,13].

Like biological maturation, age also exerts a determining influence on athletic performance in adolescent stages [14]. Changes in the hormonal environment that occur with advancing age during adolescence, both in boys and girls, seem to be the basis for the differences found by some authors in later stages [9,14,15]. The maturation process also shows differences between sexes, with girls reaching peak height velocity (PHV) at an earlier age (9–15 years old) than boys (12–16 years old) [9]. Thus, in the stages prior to puberty, no differences in physical performance or kinanthropometric variables are found between the sexes [14,16]. However, the hormonal changes that occur around the age at peak height velocity (APHV), the most notable being the increase in testosterone in boys [15], induce differences in body composition and physical performance that favor the appearance of differences between boys and girls [14].

Due to the importance of age and maturation in relation to adolescent sports, it has been observed that the traditional forms of grouping by chronological age may be a disadvantage for those players who mature later, or who were born in the last months of the year [12]. For this reason, several investigations have recently emerged addressing the relative age effect (RAE), understood as the tendency to overrepresent players born in the first months of the year in the selection process [17]. The RAE has been contrasted on several occasions in high-level adolescent players in team sports [12,18,19] and seems to be more evident around the APHV [11], obtaining better results for those players born in the first months of the year. However, in sports such as volleyball, it seems that the relationship of RAE with performance and chances of selection remain unclear, possibly due to the specific characteristics of the sport [20].

Despite the influence of age, biological maturation, and RAE on performance and kinanthropometric characteristics in adolescent athletes, little information has been found about the relationship of these variables with the differences found between sexes in volleyball players. Notwithstanding all the above, the aim of the present investigation was to analyze the differences between male and female adolescent volleyball players in kinanthropometric variables and physical fitness tests in relation to age, the maturity offset, and relative age.

## 2. Materials and Methods

### 2.1. Participants

A total of 96 1st Regional Division adolescent volleyball players, 48 males (age = 14.17 ± 1.00 years old) and 48 females (age = 14.41 ± 1.21 years old), volunteered to take part in the study. Rstudio software (3.15.0 version, Rstudio Inc., Boston, MA, USA), was used to perform the sample size calculations, setting the standard deviation (SD) based on the APHV reported in previous studies (SD = 0.65) [21]. The significance level for the calculation was set at α = 0.05. With an estimated error (d) of 0.183 years from peak height velocity, the sample size needed was 48 subjects per group. The coaches, parents, and players were informed of the measuring protocol and signed an informed consent form before starting the study. The inclusion criteria were (a) to be an under-16 (U-16) age category player, due to the chronological age cut-off points set for the competitions, (b) to play volleyball at least three times per week, (c) to take part in an official federated competition, (d) to have played volleyball at least two consecutive seasons at the time of measurements. The exclusion criteria were (a) to suffer an injury that prevented them from completing the tests, and (b) to have missed more than 25% of training sessions in the last 3 months [22]. 

### 2.2. Procedures

A cross-sectional design was followed in the present study, in accordance with the STORBE guidelines [23]. The measurement protocol was reviewed and authorized by the institutional ethics committee, in accordance with the Code of World Medical Association (Code: CE061921). The statements of the Declaration of Helsinki were followed during the entire process. The measurement protocol was registered before the start of the study at ClinicalTrials.gov (code: NCT04495595). The assessment was carried out in the players’ usual training hall. Sociodemographic and sporting information was collected from the players, and the coaches were asked to classify the players according to standardized criteria. Subsequently, a kinanthropometric assessment was carried out, followed by the implementation of physical fitness tests.

### 2.3. Socio-Demographic Questionnaire and Players’ Success Assessment

The players were asked to self-complete a questionnaire designed ad hoc, where they were asked their age and date of birth, days of training per week, years of experience playing volleyball in federated competition, days they had missed training in the last three months, and whether they had suffered any recent injury or were currently injured. A researcher explained each question in detail prior to the completion of the questionnaire and supervised the process.

At the same time, according to previous research [24], coaches were asked to rank the players in the following categories: “Leading team players”, “Other important players”, and “players who rarely enter the game”, depending on their role in the team. The number of teams in the league was divided into three groups, top-classification teams, mid-classification teams, and bottom-classification teams, with the same number of teams in each group. Using this classification, leading team players and other important players of the top-classification teams, and leading team players of the mid-classification teams were categorized as more successful (MS) players. The leading players of the bottom-classification teams, and other important players and players who rarely enter the game of the other team groups, were categorized as less successful (LS) players. This classification was performed following Katić et al.’s methods [24].

### 2.4. Kinanthropometric Measurements and Biological Maturation

The kinanthropometric measurements were performed in accordance with the protocols described by the International Society for the Advancement in Kinanthropometry (ISAK) [25]. Accredited ISAK kinanthropometrists (levels 2 and 3) performed all the measurements. All measurements were taken twice. A third measurement was taken when the difference between the first and second measurements was greater than 5% for the skinfolds or 1% for the other measurements. The mean between measurements, in the case of two attempts, and the median, in the case of three attempts, was taken as the final value. The intra- and inter-evaluator technical error of measurement (TEM) were calculated in a sub-sample. The intra-evaluator TEM was 0.09% in basic measurements, lengths, heights, and girths; and 1.07% in skinfolds; and the inter-evaluator TEM was 0.05% in basic measurements of lengths, heights, and girths; and 2.86% in skinfolds.

Four basic measurements (body mass, height, sitting height, and arm span); eight skinfolds (triceps, subscapular, biceps, iliac crest, supraspinale, abdominal, thigh and calf); six girths (arm relaxed, flexed and tensed arm, waist, hips, middle thigh and calf); five breadths (biacromial, biiliocristal, humerus, bi-styloid and femur); three lengths (acromiale-radiale, radiale-stylion, and mid-stylion dactylion); and a height (ilioespinale) were measured. A SECA 862 scale (SECA, Hamburg, Germany) with an accuracy of 100 g was used for measuring body mass; a SECA stadiometer (SECA, Hamburg, Germany) with an accuracy of 0.1 cm for measuring standing height and sitting height; an arm span meter (Smartmet, Jalisco, Mexico) with an accuracy of 0.1 cm for measuring arm span; a skinfold caliper (Harpenden, Burguess Hill, UK) with an accuracy of 0.2 mm accuracy for measuring skinfolds; an inextensible tape (Lufkin, Missouri, TX, USA) with 0.1 cm accuracy for measuring perimeters; a segmometer (CESCORF, Porto Alegre, Brazil) with 0.1 cm accuracy for measuring heights and lengths; an anthropometer (Realmet, Barcelona, Spain) and a small girth sliding caliper (Holtain, Crymych, UK) with 0.1 cm accuracy for measuring diameters. The final values of the kinanthropometric measurements were used to calculate the variables of the body mass index (BMI), fat mass [26], muscle mass [27], bone mass [28], somatotype [29], ∑6 skinfolds (triceps, subscapular, supraspinale, abdominal, thigh and calf), ∑8 skinfolds (triceps, subscapular, biceps, iliac crest, supraspinale, abdominal, thigh and calf), cormic index [(sitting height/height) * 100], relative arm span [(arm span/height) * 100], upper limb length [acromiale-radiale length + radiale-stylion length + stylion-medio dactylion length], corrected girths of the arm [arm relaxed girth − (π * triceps skinfold)], thigh [middle thigh girth − (π * thigh skinfold)] and calf [calf girth − (π * calf skinfold)], the muscle-bone index [muscle mass/bone mass] and waist to hip ratio (waist girth/hip girth).

### 2.5. Biological Maturation

Mirwald et al. (2002) sex-specific formula was used to estimate the maturity offset of the players. From the maturity offset, the APHV of each subject was calculated using the formula: APHV = chronological age − maturity offset result. This method proved to be valid for estimating the maturity offset with respect to the gold standard using regression equations with an R^2^ = 0.92–0.89 in the case of boys and an R^2^ = 0.91–0.88 in the case of girls.

### 2.6. Physical Fitness Test

All physical fitness tests were performed by two investigators familiar with the technique and with previous experience in the evaluation of physical fitness in the adolescent population. Each investigator was in charge of the same tests during the measurement sessions, in order to avoid an inter-rater error. The intraclass correlation coefficient (ICC) was 0.995 (95% confidence interval 0.989–0.997), and the coefficient of variation (CV) was 2.3%. The sit-and-reach and back scratch tests were performed before the warm-up because an unequal effect of the warm-up on performance in flexibility tests has been observed [30]. The participants then underwent a standardized warm-up supervised by the researchers, consisting of ten minutes of continuous running, articular mobility, and familiarization with the tests they were about to perform. A long jump, medicine ball throw, countermovement jump (CMJ), 20-m sprint, and agility (9-3-6-3-9) test were performed in the specified order. The selected order and test assessment was performed according to previously described protocols [21,24,31,32,33,34]. The players performed two attempts of each test, with a rest between attempts of two minutes. The mean of the two attempts made in each test was used as the final value used for the analysis.

The sit-and-reach test was performed with the Acuflex Tester III (Novel Products, Rockton, IL, USA); the back scratch test with a millimeter ruler (GIMA, Gessate, Italy); the long jump and medicine ball throw tests with a tape measure (HaeSt, Wolfenbüttel, Germany) with a 0.1 cm accuracy; the CMJ with a force platform with a sampling frequency of 200 Hz (MuscleLab, Stathelle, Norway); the sprint test (20 m) with MySprint (Apple Inc., Cupertino, CA, USA) [35]; and the agility test (9-3-6-3-9) with five photocells (Microgate, Bolzano, Italy).

### 2.7. Statistical Analysis

The distribution of the sample was analyzed using the Kolmogorov-Smirnov test. The kurtosis and skewness of the variables were calculated, as well as homogeneity using Levene’s test. A descriptive analysis of the variables measured was carried out, including the mean and standard deviation (SD). To analyze the differences between boys and girls in the continuous quantitative variables, a MANCOVA analysis was performed, introducing sex as a grouping variable and age, maturity offset and birth quartile as covariates. Both main effects and interactions between variables were tested to determine their influence on the differences found according to sex. The effect size was calculated with partial eta squared (Ƞ^2^_p_). Bonferroni’s post hoc was used to analyze differences between groups. Chi^2^ test was used to analyze differences in discrete quantitative variables and qualitative variables. The significance level was set a priori at *p* < 0.05. All statistical analyses were performed with SPSS v.23 software (IBM, Armonk, NY, USA).

## 3. Results

### 3.1. Maturity Status Results

The results of the comparison between sexes with respect to the APHV and the main effects of the covariates can be seen in Table 1. Significant differences were observed between male and female players (*p* < 0.001), with an effect of the covariates age (*p* < 0.001) and maturity offset (*p* = 0.048) in the model, but not of the covariate birth quartile. However, the analysis of the intersections between sex and the covariates (sex*age; sex*maturity offset; sex*birth quartile) did show an influence of these covariates on the differences found between groups (*p* < 0.001).

Table 2 shows the Bonferroni adjustment. The pairwise comparison showed significant differences between male and female players, including the models with the covariates (*p* < 0.001), with an earlier APHV in the female group.

### 3.2. Kinanthropometry Results

Regarding the kinanthropometric variables, significant differences were observed between sexes in the basic measures (*p* < 0.001–0.042), except in body mass; in the bone and muscle variables (*p* < 0.001–0.004); in the somatotype components (*p* < 0.001–0.040); in the skinfolds sum (*p* = 0.003–0.013); in the body composition components and in the muscle-bone index (*p* < 0.001–0.030) (Table 1). The covariate age showed main effects in bone related variables (arm span, sitting height, biacromial, and biileocrestal breadth) and in the muscle mass and percentage (*p* = 0.001–0.038). The covariate maturity offset showed main effects in all the kinanthropometric variables (*p* < 0.001–0.003), except for the somatotype components and the fat and muscle percentages. The covariate birth quartile did not demonstrate statistical significance in terms of main effects on the model (*p* < 0.05).

The analysis of the interactions between sex and age showed a significant influence of age on the differences found between groups in the basic measures (*p* < 0.001–0.007); upper limb length, biacromial, biiliocrestal, and humerus breadths (*p* < 0.001–0.031); corrected leg girth and muscle and bone masses (*p* = 0.001–0.005) (Table 2). The analysis of interactions between sex and offset maturity showed a significant influence of maturation on all kinanthropometric variables (*p* < 0.001–0.039), except somatotype, skinfolds sum, and muscle and fat percentages (Table 2). Analysis of interactions between sex and birth quartile showed a significant influence of birth quartile on all kinanthropometric variables (*p* < 0.001–0.015), except for biiliocrestal breadth, mesomorphy, fat mass, and BMI.

Pairwise comparation showed statistical differences between groups in the four models (sex, sex*age, sex*maturity offset, sex*birth quartile) (*p* < 0.001–0.030), except for the biiliocrestal breadth and the BMI, the mesomorphic component in the interaction models and the skinfolds sums, bone percentage and fat mass in the sex*age interaction (Table 3). Male players showed higher values in basic measures, bone, and muscle-related variables, whilst female players showed higher values in fat-related values (Table 3).

### 3.3. Physical Fitness Results

Physical fitness differences between male and female players and covariable main effects can be observed in Table 1. Significant sex differences were found in all physical fitness tests except for agility (*p* < 0.001–0.012). The covariate age showed a significant effect on the model in the long jump, medicine ball throw, CMJ height and power, 20 m sprint, and agility tests (*p* = 0.001–0.007). The covariate maturity offset showed statistical effects over the long jump, medicine ball thrown CMJ height, and power tests (*p* < 0.001–0.007). The covariate birth quartile showed no main effects on the model.

The interaction between variables (sex*age, sex*maturity offset, sex*birth quartile) showed that the three covariables had significant influence in the differences observed between groups in long jump, medicine ball throw, CMJ height and power (*p* < 0.001–0.026), and also in sprint and agility tests in the case of birth quartile (*p* < 0.001–0.009) (Table 2).

Pairwise comparation showed statistical differences between groups in the four models (sex, sex*age, sex*maturity offset, sex*birth quartile) in back scratch test, long jump, medicine ball throw, CMJ height and power, and 20 m sprint (*p* < 0.001–0.012). Statistical differences were also found in the sit-and-reach test in the sex model (*p* < 0.004), and in the agility test in sex*maturity offset and sex*birth quartile model (*p* = 0.002) (Table 4).

### 3.4. Chi^2^ Results

The results of the Chi^2^ analysis can be seen in Table 5. No significant differences were observed between groups in the variables “training days”, “years playing volleyball”, “years in competition” or “success”.

## 4. Discussion

One of the main objectives of the present study was to analyze the differences between boys and girls in terms of kinanthropometric variables. In this sense, significant differences were observed between groups in basic measurements, somatotype components, bone and muscle variables, and body composition components. These results are in line with previous studies that found differences between sexes in the variables studied in track and field athletes of similar age to the present study [16]. In addition, the introduction of the age covariate showed significant influence on bone variables and muscle mass and percentage, while the covariate maturity offset showed influence on the differences found in all kinanthropometric variables except somatotype and fat and muscle percentages. In this line, it has been observed that the differences between boys and girls in the early stages of pubertal development are not significant, but increase as pubertal development progresses [14,36]. Not surprisingly, the differences between boys and girls found in later stages could have their origin in the pubertal growth spurt, being especially relevant in the case of boys, since the hormonal changes that occur at this stage have a greater influence on the determinants of sports performance in them [15,16]. In the case of the present study, the population included was homogeneous in relation to age, observing a mean age of both boys and girls around 14 years, which means that while girls have passed APHV, the age of boys is close to their APHV [9]. This proximity in chronological age of the group of boys with respect to their APHV and the relationship of this with the differences between sexes [14], together with the homogeneity of the group in terms of chronological age could be the basis of the significant differences found.

When analyzing the results of the Bonferroni adjustment, it was observed that the group of boys showed higher values in both bone and muscle variables, as well as in the components of muscle and bone body composition, while the girls obtained higher results in the adipose related variables and the fat component of body composition, with no differences observed in body mass or BMI. In the case of boys, circulating testosterone increases up to 30 times with respect to values measured before the growth spurt [15]. In girls, testosterone levels also increase during puberty, but more gradually and to a lesser extent than in the male population [15], and there is also an increase in estrogen during this period [37]. The effects of testosterone on the increase in muscle and bone mass have shown to have a strong dosis-effect relationship between the increase in endogenous testosterone and the increase in muscle mass, as well as larger and denser bones [38,39]. This increase in bone and muscle variables has been related to greater production of strength and power [40], variables of vital importance in volleyball performance [6]. On the other hand, the increase in estrogen concentration has been related to adipose tissue, observing an accumulation of fat mass and its redistribution in relation to this sex hormone [41]. It could be for this reason that both age, to a lesser extent, and biological maturation could be affecting the differences found between boys and girls in the kinanthropometric variables in the present study.

Regarding the physical fitness tests, significant differences between sexes were observed without the influence of any of the covariates in the sit-and-reach and back scratch tests, where the group of girls obtained better results. These results are in line with other studies that have analyzed flexibility and range of motion (ROM) comparing between sexes [32,42]. Flexibility test performance has also been associated in the literature with age, finding that flexibility increases with advancing age, perhaps due to the susceptibility to improvement with training [32,43]. However, in the present study, no influence of age was observed in the differences found. On the other hand, the relationship of flexibility with biological maturation and birth quartile remains unclear [32], and no significant effect was found neither in the present work, so future research could try to clarify this relationship.

On the other side, differences between males and females were observed in the long jump, medicine ball throw, CMJ height and power, sprint, and agility tests, observing an interaction of age, maturity offset and birth quartile in the differences. All the tests where the differences were found are related to the ability to produce power and strength [21,34]. Among the factors that positively affect the production of muscle power, it has been observed that one of the key factors is muscle mass, with a relationship existing between the increase of muscle mass and the production of power [40,44]. Moreover, bone structure and biomechanics also play a crucial role in strength application [45]. These factors associated with better physical performance in tests requiring the application of force are favored by the increase in testosterone during adolescence [14,15]. These differences in testosterone concentration between boys and girls that appear during the growth spurt of boys around the APHV are maintained throughout adolescence [9,15], a fact that could help to understand the influence of both age and biological maturation in the differences found. On the other hand, the differences induced by the hormonal environment in the muscle, bone, and adipose variables observed could also be favoring the appearance of differences in performance.

In relation to the birth quartile, it did not show statistical main effect in the MANCOVA model with respect to the kinanthropometric and physical fitness variables, although it did show influence on the differences between groups in the interaction with sex. REA has been well documented in team sports [3,12,46]. There seems to be a clear REA in favor of those players born in the first months of the year, due in part to the differences found in both chronological age and maturation with respect to their peers born in the last months of the year in adolescent athlete populations [11,12,47]. However, the REA tends to be less determinant as age advances and the maturation process of the participants tends to equalize, becoming not determinant for sporting success as they approach adulthood [46,48]. In the present study, an influence of the REA on the differences found between male and female players in kinanthropometric variables and physical fitness tests was observed. These results are similar to those found in previous studies in both male and female volleyball players, in which significant differences were observed in relation to the birth quartile [3]. Nevertheless, the relationship of birth quartile with being chosen in a sports selection process remains unclear, since in young elite athletes a REA has not been observed as in other athlete populations [3,12,20,46,48]. This is why it is a topic that should be explored in future research with a longitudinal design.

The differences found between boys and girls could be altered by differences in training, due to the influence of systematic physical exercise on kinanthropometric variables and variables related to physical fitness tests [49,50]. It has been observed that changes in kinanthropometric variables and sports performance improvement occur during adolescence in the absence of physical exercise, but are much more marked when adolescents exercise [9,51,52]. That is why we analyzed the differences between sexes in terms of days of training, years playing volleyball, years competing, and whether they had been categorized as successful players or not. However, in this study, no significant differences were observed with respect to the volume of training measured in days, nor were differences observed in the years of experience playing volleyball or competing, nor in the number of male and female successful players.

The present study is not free of limitations. Among the most important limitations are the descriptive and cross-sectional nature of the study, a relatively small sample size, the age of the participants, which together with the design of the study does not allow us to analyze the evolution of the phenomenon studied throughout the entire maturation stage, and the method of estimating biological maturation, which although it is a widely used method in sports science [8], is not the hand and wrist X-ray, considered the gold standard for the calculation of the maturity offset [9]. Future research could evaluate the influence of age, biological maturation, and birth quartile on the differences between boys and girls in kinanthropometric and physical fitness variables in volleyball from the age of sports initiation to advanced stages of adolescence, using longitudinal designs, with a larger sample, to know the evolution of the differences throughout the growth stage.

## 5. Conclusions

There is a clear influence of age and biological maturation in the differences found between sexes in adolescent volleyball players, while the birth quartile seems to have less influence as a main effect. The male volleyball players showed higher values in the basic kinanthropometric variables; bone breadths, except biiliocrestal; corrected muscle girths; bone and muscle masses, and muscle-bone index, while female players showed higher values in adipose related variables. In addition, significant differences were observed in the physical condition tests dependent on muscle strength and power, with boys showing higher values, while girls showed better performance in the flexibility tests. The differences found in both anthropometric variables and physical fitness test performance, and the relationship of age and maturation on the results could affect volleyball performance in both girls and boys after the APHV and become more marked as adolescence progresses. The practical implications of these results, together with the rules of the sport in youth categories, could be related to the creation of mixed teams and competitions between boys and girls until differences in kinanthropometric variables and physical performance are present around the APHV, moving at later stages to splitting the competition between boys and girls when differences in these variables may be determinant in volleyball sports performance. It would also be interesting during these stages of growth to include the evaluation of biological maturation when categorizing players to establish in which category they compete, and not to use chronological age as the only criterion, as is usually done, since those with more advanced maturation will have a competitive advantage as their anthropometric variables and physical condition performance variables will be affected.

## Figures and Tables

**Table 1:** Differences between groups in maturation, kinanthropometric and physical fitness variables and covariables main effects.

Variable	Group		MANCOVA											
	Mean ± SD		Sex			Age			Maturity Offset			Birth Quartile		
	Males (n = 48)	Females (n = 49)	F	*p*	Ƞ^2^_p_	F	*p*	Ƞ^2^_p_	F	*p*	Ƞ^2^_p_	F	*p*	Ƞ^2^_p_
APHV (years)	13.58 ± 0.57	12.34 ± 0.57	115.86	<0.001	0.549	23.80	<0.001	0.234	3.27	0.048	0.051	0.08	0.772	0.001
Body mass (kg)	62.55 ± 13.25	57.79 ± 10.69	3.72	0.057	0.038	1.23	0.270	0.016	50.26	<0.001	0.392	1.762	0.188	0.022
Height (cm)	170.71 ± 9.22	163.29 ± 6.45	19.10	<0.001	0.167	2.74	0.102	0.034	66.87	<0.001	0.462	0.640	0.426	0.008
Arm spam (cm)	173.28 ± 10.20	163.88 ± 7.02	22.68	<0.001	0.193	4.44	0.038	0.054	55.81	<0.001	0.417	0.847	0.360	0.011
Sitting height (cm)	87.60 ± 4.76	85.81 ± 3.13	4.25	0.042	0.043	12.32	0.001	0.136	206.60	<0.001	0.726	0.918	0.341	0.012
Upper limb length (cm)	77.43 ± 4.32	73.07 ± 2.89	24.97	<0.001	0.208	2.80	0.099	0.035	40.32	<0.001	0.341	2.10	0.151	0.026
Biacromial breadth (cm)	37.20 ± 2.69	35.27 ± 1.69	14.68	<0.001	0.134	7.78	0.007	0.091	98.99	<0.001	0.559	2.69	0.105	0.033
Biiliocrestal breadth (cm)	26.10 ± 2.11	26.59 ± 2.12	0.95	0.331	0.010	6.21	0.015	0.074	65.55	<0.001	0.457	1.78	0.186	0.022
Femur breadth (cm)	9.88 ± 0.55	9.14 ± 0.50	45.34	<0.001	0.323	0.24	0.626	0.003	24.17	<0.001	0.237	0.01	0.914	<0.001
Humerus breadth (cm)	6.86 ± 0.40	6.37 ± 0.38	40.70	<0.001	0.300	1.07	0.304	0.014	18.02	<0.001	0.188	0.56	0.459	0.007
Bi-styloid breadth (cm)	5.32 ± 0.34	4.93 ± 0.25	38.17	<0.001	0.287	0.31	0.577	0.004	10.72	0.002	0.121	0.65	0.423	0.008
Corrected arm girth (cm)	23.00 ± 2.86	20.79 ± 2.05	17.70	<0.001	0.157	0.21	0.652	0.003	23.19	<0.001	0.229	0.58	0.449	0.007
Corrected thigh girth (cm)	44.47 ± 4.48	41.80 ± 4.34	8.50	0.004	0.082	3.51	0.065	0.043	51.59	<0.001	0.398	2.36	0.128	0.029
Corrected leg girth (cm)	31.77 ± 2.29	29.49 ± 2.90	13.61	<0.001	0.125	9.10	0.003	0.105	48.05	<0.001	0.381	2.00	0.162	0.025
Endomorphy	2.74 ± 1.65	3.94 ± 1.31	13.43	<0.001	0.124	0.20	0.660	0.002	2.14	0.148	0.027	3.79	0.055	0.046
Mesomorphy	4.57 ± 1.29	3.94 ± 1.14	6.74	0.011	0.066	0.21	0.649	0.003	2.59	0.112	0.032	0.96	0.330	0.012
Ectomorphy	3.46 ± 2.51	2.59 ± 1.28	4.35	0.040	0.044	0.00	0.955	<0.001	0.86	0.358	0.011	0.31	0.580	0.004
∑6 Skinfolds (mm)	66.65 ± 34.44	87.31 ± 25.04	9.07	0.003	0.087	0.79	0.377	0.010	2.56	0.114	0.032	2.77	0.100	0.034
∑8 Skinfolds (mm)	84.47 ± 44.51	109.07 ± 33.02	6.43	0.013	0.063	0.94	0.334	0.012	2.43	0.123	0.030	2.81	0.098	0.035
Fat mass (%)	16.81 ± 7.50	24.24 ± 5.97	26.07	<0.001	0.215	0.78	0.379	0.010	2.09	0.152	0.026	2.58	0.113	0.032
Muscle mass (%)	38.53 ± 2.59	31.06 ± 1.72	260.43	<0.001	0.733	5.87	0.018	0.070	1.74	0.190	0.022	0.00	0.949	<0.001
Bone mass (%)	18.02 ± 2.48	16.29 ± 1.95	14.12	<0.001	0.129	0.18	0.676	0.002	9.67	0.003	0.110	3.61	0.061	0.044
Fat mass (kg)	11.18 ± 7.37	14.41 ± 5.71	4.87	0.030	0.049	0.01	0.927	<0.001	10.52	0.002	0.119	2.30	0.133	0.029
Muscle mass (kg)	23.96 ± 4.56	17.94 ± 3.38	51.55	<0.001	0.352	4.50	0.037	0.055	79.13	<0.001	0.504	1.46	0.231	0.018
Bone mass (kg)	11.03 ± 1.49	9.25 ± 0.96	45.44	<0.001	0.324	1.63	0.205	0.020	54.72	<0.001	0.412	0.01	0.939	<0.001
BMI (kg /m^2^)	21.36 ± 3.63	21.59 ± 3.23	0.06	0.807	0.001	0.09	0.760	0.001	14.42	<0.001	0.156	1.56	0.216	0.020
Muscle-bone index	2.17 ± 0.27	1.94 ± 0.28	18.06	<0.001	0.160	3.33	0.072	0.041	19.14	<0.001	0.197	3.82	0.054	0.047
Sit-and-reach test (cm)	0.94 ± 8.58	5.75 ± 9.14	8.61	0.004	0.083	0.15	0.705	0.002	1.94	0.168	0.024	0.16	0.689	0.002
Back scratch test (cm)	1.60 ± 7.29	4.69 ± 4.84	6.50	0.012	0.064	0.82	0.369	0.010	2.53	0.116	0.031	0.34	0.563	0.004
Long jump (m)	1.97 ± 0.41	1.63 ± 0.20	28.22	<0.001	0.229	8.19	0.005	0.095	7.55	0.007	0.088	0.43	0.514	0.005
Medicine ball throw (m)	6.12 ± 1.43	4.94 ± 0.88	18.04	<0.001	0.160	10.09	0.002	0.115	73.54	<0.001	0.485	0.74	0.392	0.009
CMJ height (cm)	29.55 ± 6.18	24.50 ± 4.73	19.01	<0.001	0.167	13.00	0.001	0.143	7.58	0.007	0.089	0.94	0.336	0.012
CMJ power (W)	733.34 ± 166.50	613.46 ± 103.59	16.03	<0.001	0.144	7.57	0.007	0.088	97.03	<0.001	0.554	0.59	0.444	0.008
20 m sprint (s)	3.83 ± 0.27	4.19 ± 0.29	40.90	<0.001	0.301	9.83	0.002	0.112	2.89	0.093	0.036	0.00	0.989	<0.001
Agility test (s)	9.02 ± 0.72	9.21 ± 1.11	1.35	0.249	0.014	8.33	0.005	0.097	3.17	0.079	0.039	0.13	0.719	0.002

APHV: age at peak height velocity; BMI: Body mass index; CMJ: counter movement jump.

**Table 2:** Interaction between sex groups and the covariables age, maturity offset and birth quartile.

Variable	MANCOVA								
	Sex*Age			Sex*Maturity Offset			Sex*Birth Quartile		
	F	*p*	Ƞ^2^_p_	F	*p*	Ƞ^2^_p_	F	*p*	Ƞ^2^_p_
APHV (years)	1.43 × 10^18^	<0.001	1.000	1.17 × 10^18^	<0.001	1.000	42.34	<0.001	0.521
Body mass (kg)	5.28	0.007	0.113	35.35	<0.001	0.466	7.63	0.001	0.164
Height (cm)	9.95	<0.001	0.193	79.84	<0.001	0.663	30.32	<0.001	0.437
Arm spam (cm)	7.39	0.001	0.151	32.10	<0.001	0.442	29.09	<0.001	0.427
Sitting height (cm)	18.95	<0.001	0.313	317.70	<0.001	0.887	19.67	<0.001	0.335
Upper limb length (cm)	7.33	0.001	0.150	30.45	<0.001	0.429	32.63	<0.001	0.456
Biacromial breadth (cm)	12.12	<0.001	0.226	49.62	<0.001	0.551	17.31	<0.001	0.307
Biiliocrestal breadth (cm)	6.76	0.002	0.140	36.19	<0.001	0.472	1.96	0.148	0.048
Femur breadth (cm)	0.44	0.644	0.011	9.13	<0.001	0.184	17.76	<0.001	0.313
Humerus breadth (cm)	3.62	0.031	0.080	11.51	<0.001	0.221	33.97	<0.001	0.466
Bi-styloid breadth (cm)	1.61	0.207	0.037	3.38	0.039	0.077	17.49	<0.001	0.310
Corrected arm girth (cm)	2.89	0.061	0.065	12.42	<0.001	0.235	14.88	<0.001	0.276
Corrected thigh girth (cm)	2.99	0.056	0.067	12.30	<0.001	0.233	7.61	0.001	0.163
Corrected leg girth (cm)	6.87	0.002	0.142	18.74	<0.001	0.316	16.74	<0.001	0.300
Endomorphy	0.13	0.880	0.003	0.73	0.486	0.018	10.57	<0.001	0.213
Mesomorphy	0.03	0.971	0.001	0.01	0.990	<0.001	0.97	0.383	0.024
Ectomorphy	0.08	0.923	0.002	0.08	0.921	0.002	3.69	0.029	0.086
∑6 Skinfolds (mm)	0.13	0.882	0.003	1.53	0.223	0.036	7.17	0.001	0.155
∑8 Skinfolds (mm)	0.10	0.901	0.002	1.48	0.233	0.035	6.52	0.002	0.143
Fat mass (%)	0.53	0.592	0.013	2.10	0.129	0.049	15.39	<0.001	0.283
Muscle mass (%)	1.18	0.312	0.028	0.70	0.497	0.017	55.10	<0.001	0.586
Bone mass (%)	2.35	0.101	0.054	4.70	0.012	0.104	4.81	0.011	0.110
Fat mass (kg)	0.75	0.477	0.018	6.05	0.004	0.130	2.68	0.075	0.064
Muscle mass (kg)	7.24	0.001	0.148	39.68	<0.001	0.495	31.39	<0.001	0.446
Bone mass (kg)	5.59	0.005	0.119	47.32	<0.001	0.539	30.72	<0.001	0.441
BMI (kg/m^2^)	0.84	0.433	0.020	4.47	0.014	0.099	0.80	0.455	0.020
Muscle-bone index	1.63	0.202	0.038	3.67	0.030	0.083	14.57	<0.001	0.272
Sit-and-reach test (cm)	1.16	0.319	0.027	1.51	0.228	0.036	1.68	0.193	0.041
Back scratch test (cm)	0.06	0.945	0.001	0.23	0.796	0.006	1.94	0.150	0.047
Long jump (m)	3.83	0.026	0.085	4.51	0.014	0.100	11.34	<0.001	0.225
Medicine ball throw (m)	16.06	<0.001	0.279	38.26	<0.001	0.486	15.94	<0.001	0.290
CMJ height (cm)	4.85	0.010	0.105	5.22	0.007	0.114	10.50	<0.001	0.212
CMJ power (W)	12.53	<0.001	0.232	49.24	<0.001	0.549	15.91	<0.001	0.290
20 m sprint (s)	2.80	0.067	0.063	2.41	0.097	0.056	14.36	<0.001	0.269
Agility test (s)	2.30	0.106	0.053	3.07	0.052	0.070	4.97	0.009	0.113

*: model including the interaction between the variables expressed; APHV: age at peak height velocity; BMI: Body mass index; CMJ: counter movement jump.

**Table 3:** Post hoc Bonferroni adjustment for the different covariates for the APHV and kinanthropometric variables.

Variable	Sex			Sex*Age			Sex*Maturity Offset			Sex*Birth Quartile		
	Mean Diff ± SD	*p*	95%CI	Mean Diff ± SD	*p*	95%CI	Mean Diff ± SD	*p*	95%CI	Mean Diff ± SD	*p*	95%CI
APHV (years)	1.24 ± 0.12	<0.001	1.01 to 1.47	1.01 ± 0.15	<0.001	0.72 to 1.30	1.43 ± 0.13	<0.001	1.17 to 1.68	1.24 ± 0.13	<0.001	0.97 to 1.50
Body mass (kg)	4.68 ± 2.43	0.057	−0.14 to 9.49	9.59 ± 3.27	0.004	3.07 to 16.10	12.74 ± 2.05	<0.001	8.66 to 16.82	9.08 ± 2.49	<0.001	4.12 to 14.04
Height (cm)	7.05 ± 1.61	<0.001	3.85 to 10.25	13.12 ± 2.01	<0.001	9.12 to 17.12	15.78 ± 1.20	<0.001	13.40 to 18.16	12.68 ± 1.64	<0.001	9.41 to 15.95
Arm spam (cm)	8.79 ± 1.84	<0.001	5.12 to 12.45	13.50 ± 2.29	<0.001	8.94 to 18.05	17.12 ± 1.43	<0.001	14.28 to 19.97	13.95 ± 1.85	<0.001	10.27 to 17.63
Sitting height (cm)	1.71 ± 0.83	0.042	0.06 to 3.35	4.28 ± 1.02	<0.001	2.26 to 6.31	6.88 ± 0.46	<0.001	5.97 to 7.79	5.30 ± 0.86	<0.001	3.59 to 7.01
Upper limb length (cm)	4.00 ± 0.80	<0.001	2.41 to 5.59	6.16 ± 0.97	<0.001	4.22 to 8.10	7.42 ± 0.64	<0.001	6.14 to 8.69	6.11 ± 0.77	<0.001	4.58 to 7.65
Biacromial breadth (cm)	1.79 ± 0.47	<0.001	0.86 to 2.72	2.41 ± 0.62	<0.001	1.17 to 3.64	3.68 ± 0.34	<0.001	3.00 to 4.37	2.81 ± 0.50	<0.001	1.82 to 3.81
Biiliocrestal breadth (cm)	−0.41 ± 0.42	0.331	−1.25 to 0.43	0.13 ± 0.51	0.801	−0.88 to 1.14	1.06 ± 0.31	0.001	0.44 to 1.68	0.55 ± 0.39	0.159	−0.22 to 1.33
Femur breadth (cm)	0.74 ± 0.11	<0.001	0.52 to 0.95	0.85 ± 0.15	<0.001	0.55 to 1.15	0.93 ± 0.11	<0.001	0.72 to 1.14	0.72 ± 0.12	<0.001	0.48 to 0.96
Humerus breadth (cm)	0.51 ± 0.08	<0.001	0.35 to 0.66	0.67 ± 0.10	<0.001	0.47 to 0.87	0.76 ± 0.07	<0.001	0.61 to 0.90	0.65 ± 0.08	<0.001	0.49 to 0.80
Bi-styloid breadth (cm)	0.38 ± 0.06	<0.001	0.26 to 0.50	0.40 ± 0.09	<0.001	0.23 to 0.57	0.44 ± 0.06	<0.001	0.31 to 0.57	0.38 ± 0.06	<0.001	0.25 to 0.51
Corrected arm girth (cm)	2.12 ± 0.50	<0.001	1.12 to 3.12	3.34 ± 0.70	<0.001	1.94 to 4.73	3.69 ± 0.49	<0.001	2.71 to 4.66	2.91 ± 0.54	<0.001	1.83 to 3.98
Corrected thigh girth (cm)	2.56 ± 0.88	0.004	0.82 to 4.30	3.43 ± 1.27	0.008	0.91 to 5.96	5.25 ± 0.80	<0.001	3.66 to 6.85	3.53 ± 0.99	0.001	1.55 to 5.50
Corrected leg girth (cm)	1.95 ± 0.53	<0.001	0.90 to 3.00	2.80 ± 0.68	<0.001	1.45 to 4.15	4.25 ± 0.45	<0.001	3.35 to 5.15	3.17 ± 0.57	<0.001	2.04 to 4.30
Endomorphy	−1.08 ± 0.29	<0.001	−1.66 to −0.49	−1.16 ± 0.44	0.010	−2.05 to −0.28	−1.25 ± 0.35	0.001	−1.94 to −0.55	−1.32 ± 0.31	<0.001	−1.94 to −0.69
Mesomorphy	0.65 ± 0.25	0.011	0.15 to 1.14	0.29 ± 0.32	0.374	−0.35 to 0.93	0.40 ± 0.25	0.114	−0.10 to 0.90	0.23 ± 0.24	0.344	−0.25 to 0.70
Ectomorphy	0.84 ± 0.40	0.040	0.04 to 1.64	1.09 ± 0.58	0.063	−0.06 to 2.24	1.08 ± 0.46	0.021	0.17 to 2.00	1.13 ± 0.42	0.009	0.29 to 1.97
∑6 Skinfolds (mm)	−17.98 ± 5.97	0.003	−29.84 to −6.13	−16.18 ± 9.02	0.077	−34.14 to 1.77	−20.31 ± 7.10	0.005	−34.45 to −6.16	−22.19 ± 6.46	0.001	−35.05 to −9.32
∑8 Skinfolds (mm)	−20.15 ± 7.95	0.013	−35.92 to −4.37	−18.19 ± 11.46	0.117	−41.02 to 4.63	−24.03 ± 9.04	0.010	−42.04 to −6.03	−26.57 ± 8.23	0.002	−42.95 to −10.20
Fat mass (%)	−6.88 ± 1.35	<0.001	−9.56 to −4.21	−6.84 ± 1.99	0.001	−10.80 to −2.89	−7.76 ± 1.57	<0.001	−10.89 to −4.64	−7.65 ± 1.43	<0.001	−10.50 to −4.80
Muscle mass (%)	7.22 ± 0.45	<0.001	6.33 to 8.11	7.09 ± 0.73	<0.001	5.65 to 8.54	8.21 ± 0.59	<0.001	7.03 to 9.39	6.89 ± 0.66	<0.001	5.58 to 8.19
Bone mass (%)	1.68 ± 0.45	<0.001	0.79 to 2.57	1.17 ± 0.64	0.070	−0.10 to 2.44	0.91 ± 0.48	0.061	−0.04 to 1.86	1.13 ± 0.45	0.015	0.22 to 2.03
Fat mass (kg)	−2.90 ± 1.31	0.030	−5.50 to −0.29	−1.86 ± 1.93	0.339	−5.70 to 1.98	−1.66 ± 1.44	0.254	−4.53 to 1.21	−2.46 ± 1.39	0.080	−5.23 to 0.30
Muscle mass (kg)	5.85 ± 0.81	<0.001	4.23 to 7.46	7.37 ± 1.08	<0.001	5.23 to 9.52	9.12 ± 0.62	<0.001	7.88 to 10.36	7.15 ± 0.91	<0.001	5.32 to 8.97
Bone mass (kg)	1.73 ± 0.26	<0.001	1.22 to 2.24	2.32 ± 0.34	<0.001	1.64 to 2.99	2.68 ± 0.21	<0.001	2.27 to 3.10	2.17 ± 0.28	<0.001	1.62 to 2.72
BMI (kg/m^2^)	−0.17 ± 0.69	0.807	−1.53 to 1.20	0.23 ± 0.95	0.813	−1.67 to 2.13	0.58 ± 0.70	0.411	−0.81 to 1.97	−0.01 ± 0.70	0.986	−1.40 to 1.38
Muscle-bone index	0.23 ± 0.05	<0.001	0.12 to 0.33	0.29 ± 0.07	<0.001	0.15 to 0.43	0.38 ± 0.05	<0.001	0.28 to 0.49	0.28 ± 0.06	<0.001	0.17 to 0.39

*: model including the interaction between the variables expressed; APHV: age at peak height velocity; BMI: Body mass index.

**Table 4:** Post hoc Bonferroni adjustement for the different covariates for the physical fitness variables.

Variable	Sex			Sex*Age			Sex*Maturity Offset			Sex*Birth Quartile		
	Mean Diff ± SD	*p*	95%CI	Mean Diff ± SD	*p*	95%CI	Mean Diff ± SD	*p*	95%CI	Mean Diff ± SD	*p*	95%CI
Sit-and-reach test (cm)	−5.21 ± 1.78	0.004	−8.74 to −1.69	−3.86 ± 2.27	0.093	−8.38 to 0.66	−3.17 ± 1.79	0.080	−6.73 to 0.39	−3.01 ± 1.67	0.076	−6.35 to 0.32
Back scratch test (cm)	−3.16 ± 1.24	0.012	−5.62 to −0.70	−4.59 ± 1.75	0.011	−8.08 to −1.10	−3.48 ± 1.38	0.014	−6.23 to −0.73	−2.46 ± 1.32	0.066	−5.08 to 0.17
Long jump (m)	0.34 ± 0.06	<0.001	0.21 to 0.47	0.23 ± 0.09	0.013	0.05 to 0.41	0.40 ± 0.07	<0.001	0.26 to 0.55	0.34 ± 0.07	<0.001	0.20 to 0.48
Medicine ball throw (m)	1.06 ± 0.25	<0.001	0.57 to 1.56	1.10 ± 0.30	<0.001	0.50 to 1.70	1.78 ± 0.18	<0.001	1.42 to 2.15	1.38 ± 0.25	<0.001	0.88 to 1.87
CMJ height (cm)	4.80 ± 1.10	<0.001	2.61 to 6.98	3.36 ± 1.39	0.018	0.60 to 6.12	6.60 ± 1.14	<0.001	4.33 to 8.87	5.18 ± 1.15	<0.001	2.90 to 7.46
CMJ power (W)	114.36 ± 28.57	<0.001	57.65 to 171.07	149.99 ± 35.95	<0.001	78.41 to 221.56	222.93 ± 20.02	<0.001	183.08 to 262.79	166.19 ± 29.85	<0.001	106.76 to 225.61
20 m sprint (s)	−0.35 ± 0.06	<0.001	−0.46 to −0.24	−0.24 ± 0.08	0.003	−0.40 to −0.09	−0.40 ± 0.06	<0.001	−0.53 to −0.27	−0.34 ± 0.06	<0.001	−0.46 to −0.21
Agility test (s)	−0.21 ± 0.18	0.249	−0.57 to 0.15	−0.15 ± 0.18	0.409	−0.52 to 0.21	−0.49 ± 0.15	0.002	−0.79 to −0.19	−0.44 ± 0.14	0.002	−0.72 to −0.16

*: model including the interaction between the variables expressed; CMJ: counter movement jump.

**Table 5:** Differences between males and females in training and selection variables.

Variable		Sex		Chi^2^ Value	*p*
		Male	Female		
Training days	3	36 (75%)	41 (85.42%)	3.702	0.296
	4	11(22.92%)	6 (12.50%)		
	5	1 (2.08%)	1 (2.08%)		
Years playing volleyball	2	16 (33.33%)	11 (22.92%)	8.993	0.174
	3	14 (29.17%)	12 (24.49%)		
	4	6 (12.5%)	11 (22.92%)		
	5	9 (18.75%)	4 (8.33%)		
	6	1 (2.08%)	2 (4.16%)		
	7	1 (2.08%)	5 (10.42%)		
	8	1 (2.08%)	3 (6.25%)		
Years in competition	1	18 (37.5%)	14 (29.17%)	5.310	0.505
	2	8 (16.67%)	6 (12.50%)		
	3	14 (29.17%)	14 (29.17%)		
	4	5 (10.42%)	5 (10.42%)		
	5	3 (6.25%)	5 (10.42%)		
	7	0 (0%)	2 (4.16%)		
	8	0 (0%)	2 (4.16%)		
Success	More successful	21 (43.75%)	20 (41.67%)	0.086	0.838
	Less successful	27 (56.25%)	28 (58.33%)		

## Data Availability

The datasets are available from the corresponding author on reasonable request.
